# Mortui vivos docent: a modern revival of temporal bone plug harvests

**DOI:** 10.3389/fnins.2023.1242831

**Published:** 2023-10-11

**Authors:** Varun Sagi, Nikitha Kosaraju, Lindsay S. Moore, Jip Y. Mulders, Mehmet Solyali, Xiaojie Ma, Donald P. Regula, Jody E. Hooper, Konstantina M. Stankovic

**Affiliations:** ^1^Department of Otolaryngology-Head and Neck Surgery, Stanford University School of Medicine, Stanford, CA, United States; ^2^David Geffen School of Medicine at UCLA, Los Angeles, CA, United States; ^3^Department of Physics, Stanford University School of Humanities and Sciences, Stanford, CA, United States; ^4^Department of Otolaryngology – Head and Neck Surgery, Massachusetts Eye and Ear and Harvard Medical School, Boston, MA, United States; ^5^Department of Pathology, Stanford University School of Medicine, Stanford, CA, United States; ^6^Department of Neurosurgery, Stanford University School of Medicine, Stanford, CA, United States; ^7^Wu Tsai Neurosciences Institute, Stanford University, Stanford, CA, United States

**Keywords:** human temporal bone, temporal bone plug cutter, microwave decalcification, histopathology, otic capsule, inner ear, otology

## Abstract

Human temporal bones (HTBs) are invaluable resources for the study of otologic disorders and for evaluating novel treatment approaches. Given the high costs and technical expertise required to collect and process HTBs, there has been a decline in the number of otopathology laboratories. Our objective is to encourage ongoing study of HTBs by outlining the necessary steps to establish a pipeline for collection and processing of HTBs. In this methods manuscript, we: (1) provide the design of a temporal bone plug sawblade that can be used to collect specimens from autopsy donors; (2) establish that decalcification time can be dramatically reduced from 9 to 3 months if ethylenediaminetetraacetic acid is combined with microwave tissue processing and periodic bone trimming; (3) show that serial sections of relatively-rapidly decalcified HTBs can be successfully immunostained for key inner ear proteins; (4) demonstrate how to drill down a HTB to the otic capsule within a few hours so that subsequent decalcification time can be further reduced to only weeks. We include photographs and videos to facilitate rapid dissemination of the developed methods. Collected HTBs can be used for many purposes, including, but not limited to device testing, imaging studies, education, histopathology, and molecular studies. As new technology develops, it is imperative to continue studying HTBs to further our understanding of the cellular and molecular underpinnings of otologic disorders.

## Introduction

Human temporal bones (HTBs) are invaluable in the study of auditory and vestibular disorders. The pathologic basis of many common otologic disorders, including Ménière’s disease and otosclerosis, was identified through the careful *post-mortem* histological analysis of HTBs (Hallpike and Cairns, 1938; Mudry, 2006). Despite their historical importance, resources directed towards the study of HTBs have decreased over time due to the high operational costs of running HTB laboratories (Chole, 2010; Monsanto et al., 2018). Today, only three otopathology laboratories remain fully operational in the United States, down from a peak of 28 in the 1980s (Monsanto et al., 2018). The closure of these laboratories has consolidated the expertise required to collect, process, and analyze HTBs to a select few institutions. Given that many steps along this pathway are time consuming and technically complex, proficiency is often achieved only after repetitive practice.

Despite these challenges, continued study of HTBs is needed to further our understanding of the pathophysiology underlying otologic disorders. Currently, only 22 of the nearly 200 deafness causing genes have reported histopathological findings (Bommakanti et al., 2019). The scarcity of histopathological knowledge also extends to patients who have undergone routine surgical procedures (Monsanto et al., 2018). HTBs from donors with well-documented medical history aid in the understanding of unknown disease pathology and serve to evaluate and improve commonly performed operations. Moreover, as innovation efforts continue to progress, HTBs serve as unmatched three-dimensional anatomic models for device development. With modern technology expanding, the multi-utility of HTBs extends even beyond these applications, and therefore, a modern update of the collection and processing methods may encourage an increased use of HTBs in otologic research.

An excellent reference titled “Techniques for Human Temporal Bone Removal: Information for the Scientific Community,” was written in 1996 (Nadol, 1996). While this manuscript is a rich source of information for collecting HTBs, certain tools listed in the manuscript, namely the temporal bone plug cutter, are no longer commercially available. The temporal bone plug cutter is specifically designed to collect a specimen which includes the inner ear, the internal auditory canal (IAC), the medial portion of the external auditory canal (EAC), tympanic membrane (TM), and a portion of the sigmoid sinus. Advantages of this approach include ease of training new users, efficient specimen collection, and minimal cosmetic defects to the donor body.

Our objective is to provide an updated resource that covers both collection and processing of HTBs to facilitate study of HTBs and accelerate progress in development of therapeutics for auditory and vestibular disorders. We outline the information necessary to manufacture a temporal bone plug saw and to collect and process HTBs for desired downstream application. Our methods emphasize affordability and flexibility by using common equipment found in laboratories, commercially available reagents, skills that can be developed in a time efficient manner, and provision of multiple options for processing that can be chosen based on project timeline and personnel skill. We also highlight recent advances in applications for HTBs to emphasize their ongoing value to the field.

## Materials and methods

### Equipment for extraction of human temporal bones

**Figure 1:**
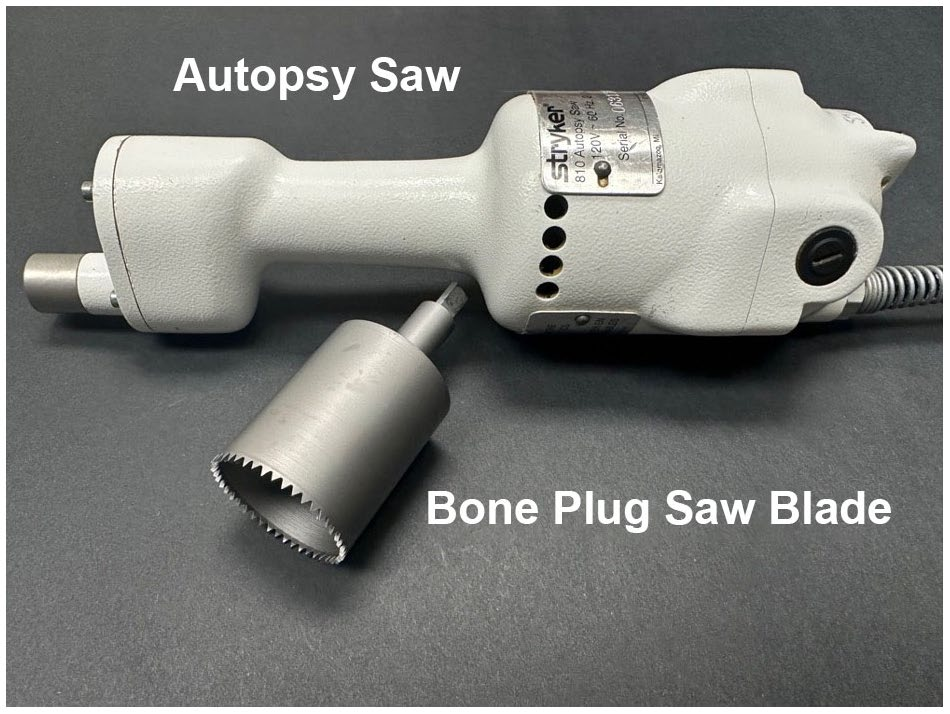
Autopsy saw and bone plug sawblade. The autopsy saw is used to drive a custom-designed bone plug sawblade for extraction of temporal bone plugs.

The equipment utilized for temporal bone plug removal includes an oscillating autopsy saw and a custom-made temporal bone plug cutter (Figure 1). Once the cutter is attached to the shaft of the saw, the connection is tightened using an 1/8 inch Allen wrench.

Detachment of the temporal bone plug from soft tissue attachments was done by using a combination of forceps and surgical scissors (straight and curved). Additional equipment that may be helpful includes scalpels, towels, and containers to hold the bone plugs.

### Partnership with pathology

To establish a successful temporal bone collection program, it is critical to form a close partnership with the Pathology department, specifically, the Autopsy service team. These colleagues can serve as advocates for temporal bone research and aid in the identification of suitable donors. They may also oversee collection procedures to ensure that they are in line with the wishes of the donor family. Additionally, they serve as excellent resources for navigating institutional guidelines surrounding the use of autopsy specimens in research.

### Bone plug extraction

**Figure 2:**
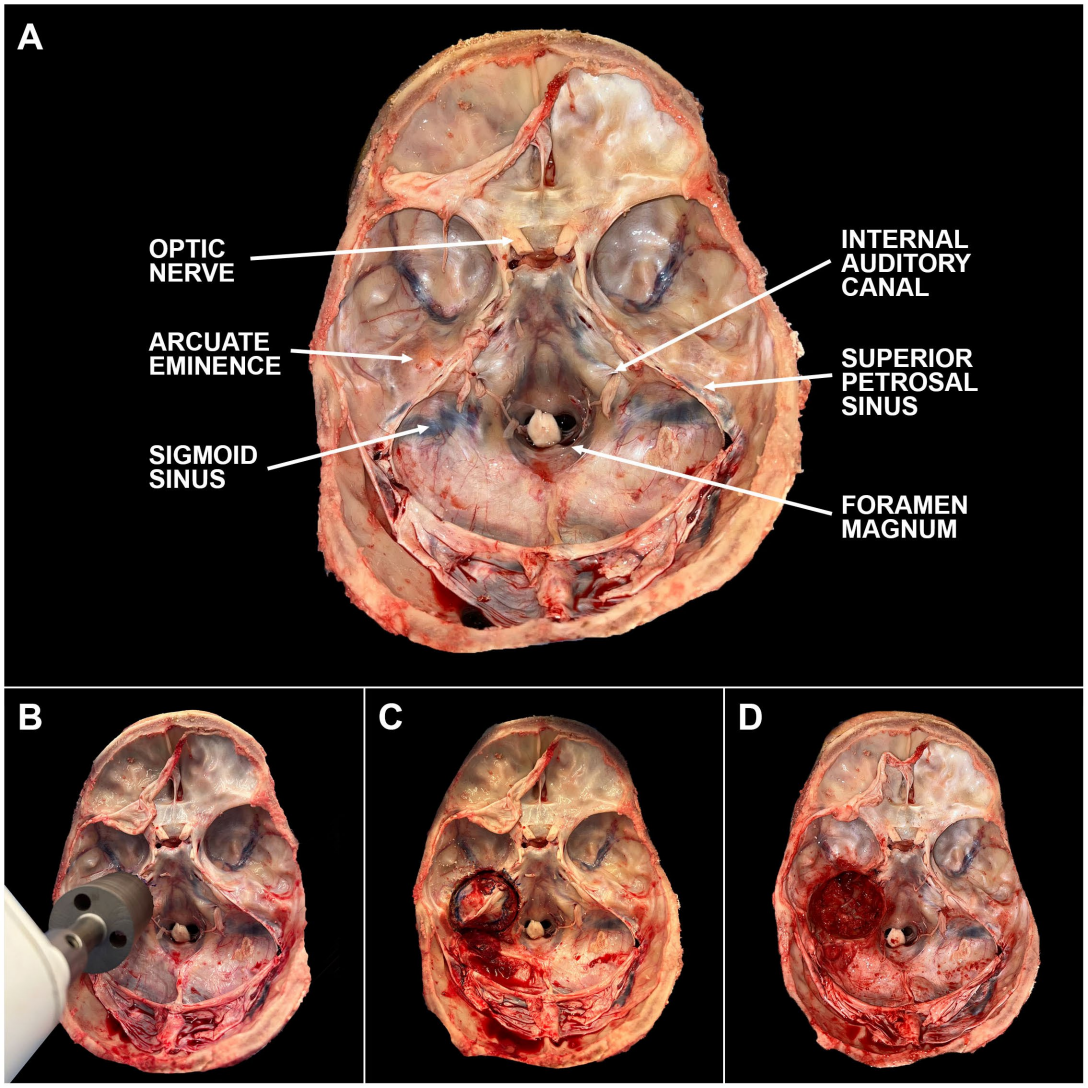
Steps of temporal bone plug extraction. **(A)** The skull base is labeled with key anatomic landmarks. **(B)** The bone plug sawblade is centered over the arcuate eminence with the internal auditory canal included in the posteromedial edge of the blade margin. Blade should be advanced in a plane perpendicular to the middle cranial fossa. Once a change in resistance is encountered, the sawblade should be removed. **(C)** Bone plug will remain in place due to inferior soft tissue attachments. These attachments can be cut using surgical scissors or a scalpel. **(D)** Bone plug has been removed from the skull base. Careful attention should be paid to minimize cosmetic defects based on final preferences of the donor and/or donor’s family. A video depicting this process can be found through the linked (Supplementary Video 1).

Optimal timing for temporal bone plug extraction occurs immediately after the autopsy team removes the brain. For ideal preservation of structures, the vestibulocochlear nerve should be cut as close to the brainstem as possible with minimal stretching of the nerve. Once the brain and brainstem have been removed, the IAC and arcuate eminence should be identified (Figure 2 and Supplementary Video 1). The bone plug sawblade is centered on the arcuate eminence such that the IAC is included at the posteromedial aspect of the saw margin. Prior to advancing the saw, an assistant should firmly grasp and stabilize the head. The saw is advanced forward in a plane that is perpendicular to the middle fossa of the skull base until a change in resistance is met when transitioning from bone to soft tissue. It can be helpful to rotate the saw back and forth while drilling to aid with advancement. Of note, it is very important to drill at the correct angle to prevent excess force on the sawblade and prevent fracturing of the blade. If it is difficult to advance the saw, it often means the sawblade is angled incorrectly and adjustments are needed. Irrigation with saline can also be helpful with thicker bone. Once a change in resistance is met, the saw is removed. To release the bone plug from its soft tissue attachments, it is firmly grasped using forceps and rotated to create room for curved surgical scissors to maneuver below the plug and cut the soft tissue attachments. Collected bone plugs can be placed in fixative or phosphate-buffered saline (PBS) depending on downstream application. Bone plugs undergoing fixation were placed in 10% neutral buffered formalin (NBF; Sigma-Aldrich, SKU HT501640) at room temperature (RT) for at least seven days. Donor demographics and clinical histories were collected using the electronic health record. Post-mortem interval (PMI) at time of collection was recorded.

### Decalcification

Following fixation in 10% NBF, bone plugs were rinsed in distilled water, and then placed in glass jars (Ted Pella, Inc., Product No. 36124) containing 0.27 M ethylenediaminetetraacetic acid (EDTA; pH 7.3; Fisher Scientific, Catalog No. S311-3). Samples were then transferred to a microwave tissue processor (PELCO BioWave^®^ Pro+, Ted Pella, Inc., Redding, CA) which had an accompanying water cooling pump (PELCO Steady Temp^™^ Pro, Ted Pella, Inc., Redding, CA). Inside the microwave, sample jars were positioned in a processing container which had circulating cooling water. Several samples were decalcified in parallel. Microwave was set to 700 watts (max power) with steady state sample temperature, measured via probe placed in one of the sample jars, set at 25°C. Decalcification was monitored radiographically through the use of micro computed tomography (micro-CT). Alternative approaches to verifying decalcification include X-rays or tapping the bone using a fine instrument such as a Rosen ear needle. If the bone depresses as cartilage depresses when tapped, decalcification has been achieved.

**Figure 3:**
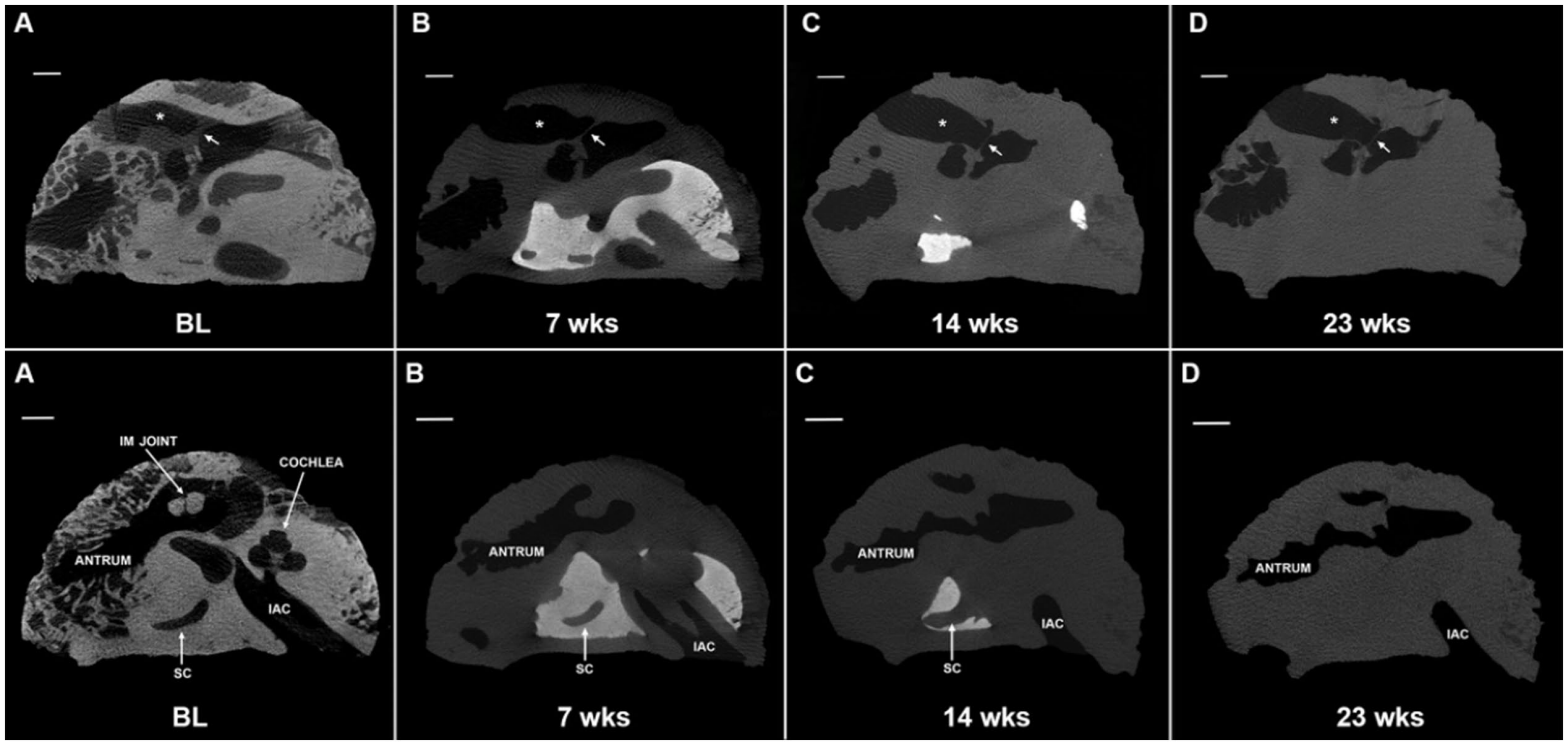
Progression of microwave and EDTA decalcification in extracted temporal bone plug. Micro computed tomography (micro-CT) scans of extracted temporal bone plugs were performed at regular intervals during decalcification. Top row: external auditory canal (*) and tympanic membrane (arrow) are labeled for orientation. Full decalcification was achieved at 23 weeks **(D)**. Bottom row: the cochlea is largely decalcified by the 7 weeks timepoint **(B)** and is completely decalcified by 14 weeks **(C)**. Calcified bone surrounding the semicircular canal (SC) is persistent through 14 weeks **(C)** with full decalcification achieved at 23 weeks **(D)**. EDTA, ethylenediaminetetraacetic acid; IAC, internal auditory canal; IM joint, incudomalleolar joint; SC, semicircular canal; BL, baseline. All scales = 5 mm.

Micro-CT images were collected at baseline (prior to EDTA initiation) and at one-to-two week intervals thereafter (Figure 3). EDTA was replaced twice weekly until end-point of decalcification was achieved. Samples were microwaved continuously with the exception of removal for EDTA changes and imaging. Once decalcification was complete, the samples were removed from the microwave and stored in fresh EDTA at RT. Samples were then monitored for microbial growth at monthly intervals, but no further EDTA changes were made unless necessary.

### Trimming

**Figure 4:**
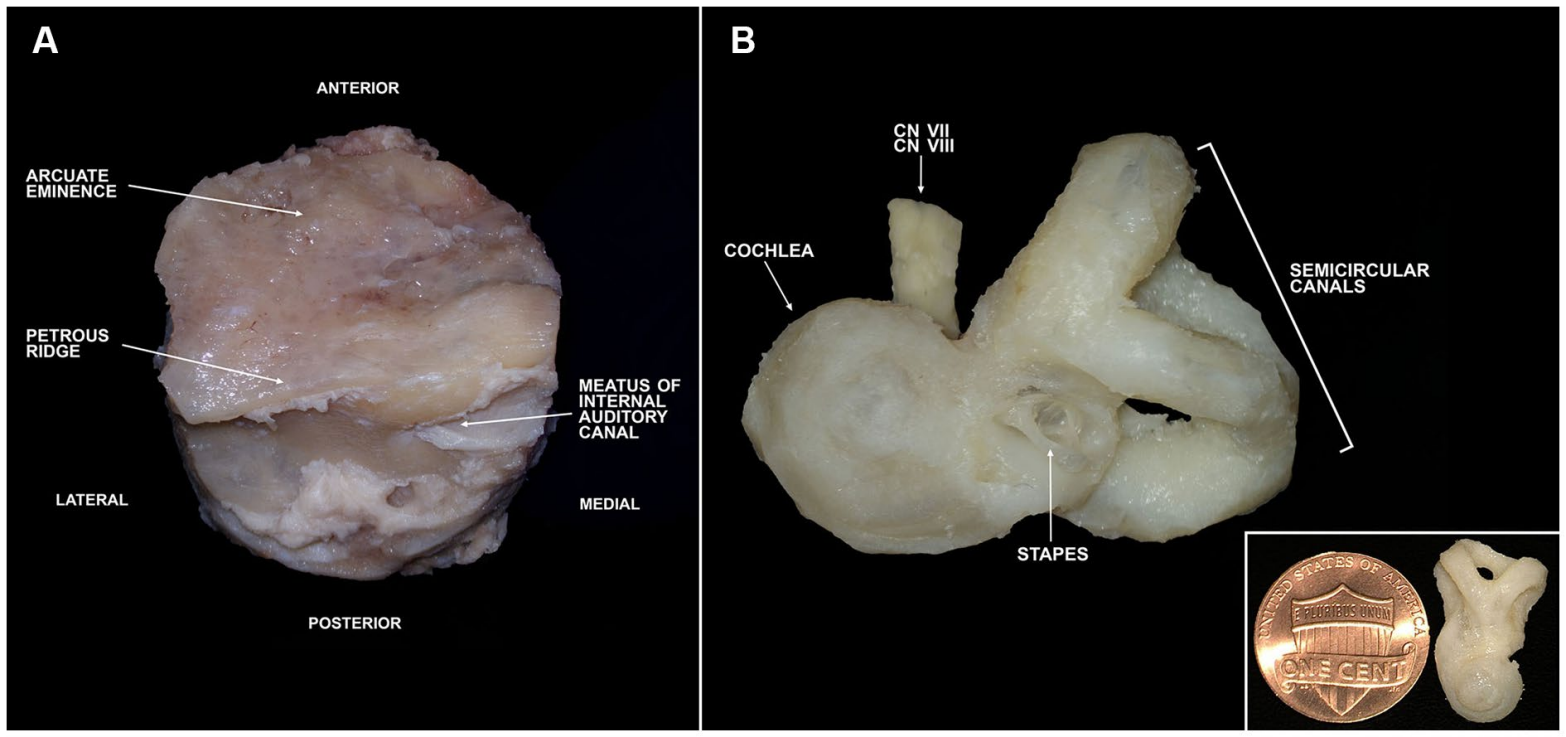
Trimming of a temporal bone plug to the otic capsule. **(A)** Full bone plug labeled with relevant anatomy following fixation in 10% neutral buffered formalin. **(B)** Fully trimmed bone plug down to the otic capsule. Inlet image in lower right shows otic capsule size in comparison to a penny. CN, cranial nerve.

To assess whether periodic trimming of bone could accelerate decalcification, a portion of fixed bone plugs underwent bone trimming sessions twice a month. Decalcified bone and soft tissues were removed under a surgical microscope by a trained medical student (NK) and a neurotology fellow (LSM) using a No. 10 or No. 15 scalpel and rongeurs with the goal of trimming down to the otic capsule (Figure 4). Trimming sessions were periodic to allow for decalcification to occur in between sessions to soften the bone enough to be able to pick it out with a scalpel or rongeur. Trimming of the bone is an alternative to drilling. If a drilling set-up is accessible, drilling to the otic capsule is a quicker method of accelerating decalcification (Supplementary Video 2), because drilling can be completed within several hours.

Successful trimming of temporal bone plugs down to the otic capsule was achieved using anatomic landmarks (Supplemental materials). The initial trimming session primarily consisted of removal of soft tissues such as the dura, blood vessels, muscular attachments, fat, and nerves (except for those exiting the IAC). The second trimming session resulted in removal of portions of the mastoid and middle ear. An effective and safe way to trim at this stage is to remove any structures lateral to the tympanic membrane. Once that is completed, an opening to the middle ear must be identified and any structures lateral to the stapes can be removed. The remaining sessions consist of whittling down to the otic capsule. The superior and lateral aspects of the specimen (orient the bone using the IAC) can be slowly shaved away until the semicircular canals (SCs) are encountered. To find the cochlea, the boundaries of the IAC are gradually trimmed. Once the cochlea and SCs have been identified, all remaining surrounding bone can slowly be removed. Delineation of the cochlear apex at this stage can be helpful for orienting the bone in downstream applications.

### Paraffin embedding for histopathology

Following decalcification, temporal bones may be processed for histopathology. Several embedding options exist, but we elected to outline paraffin embedding due to its lower equipment, technical, and cost requirements when compared to other approaches, as well as its compatibility with subsequent immunohistochemistry. One limitation of paraffin embedding is that full-size temporal bone plugs cannot be cut due to the mounting block and cutting blade size restrictions of a standard microtome. Therefore, the bone plug should either be drilled or trimmed to include only the cochlear and vestibular labyrinths. The oval and round windows should be opened to allow for paraffin to infiltrate into the inner ear.

With an appropriately sized specimen, tissue processing can be initiated. Broadly, the specimen is dehydrated through increasing concentrations of ethanol, cleared with xylene, and then embedded in paraffin. All processing steps were of one-hour durations unless otherwise stated. The sample was first placed in 80% ethanol (Fisher Scientific, Catalog No. BP28184) overnight. The next day consisted of three changes of 95% ethanol, three changes of 100% ethanol, and two changes of equal parts 100% ethanol and xylene (Fisher Scientific, Catalog No. X3P1GAL). The bone was then placed in a third change of equal parts 100% ethanol and xylene overnight. The final day included two changes of xylene, one change of equal parts xylene and paraffin (Surgipath Paraplast, Leica, Product No. 39601006), and two changes of paraffin. The sample was then embedded in a metal mold. If a mid-modiolar cut of the cochlea is desired, the sample should be embedded such that the cutting surface is parallel to the long axis of the otic capsule with the cochlea positioned upright.

The sample was sectioned using a manual microtome (Leica RM2125 RTS). Serial sections were cut at a thickness of 5–8 μm and mounted on Superfrost Plus slides (Fisher Scientific, Catalog No. 12-550-15). Slides were dried at RT for at least 1 h and then baked at 62°C for 2 h. Dried sections were stored at RT until time of staining. Sections were first deparaffinized and hydrated through three changes of xylene for 2 min each, three changes of 100% ethanol for 2 min each, one change of 70% ethanol for 1 min, one change of 50% ethanol for 1 min, and two changes of distilled water (DW) for 3 min each.

H&E staining of every fifth section was done to locate mid-modiolar sections containing all cochlear turns. Staining consisted of hematoxylin (Fisher Scientific, Catalog No. 22-220-101) for 10 min, running tap of DW for 3 min, bluing agent (Fisher Scientific, Catalog No. 22-220-106) for 1 min, running tap of DW for 3 min, eosin (Fisher Scientific, Catalog No. 22-220-104) for 1 min, and running tap of DW for 3 min. Sections were then dehydrated by placement in 70% ethanol for 1 min, three changes of 100% ethanol for 2 min each, and two changes of xylene for 2 min each. Slides were immediately mounted with Vectashield mounting medium (Vector Labs, Catalog No. H-1000). Cellular morphology was then assessed using light microscopy.

Mid-modiolar sections were used for immunohistochemistry. Slides were first deparaffinized and hydrated using the steps outlined above. Antigen retrieval was then performed using heat-induced epitope retrieval. Slides were placed in a vertical slide rack with a tight-fitting snap closure that contained citrate buffer, pH 6.0 (Sigma-Aldrich, SKU C9999-100ML). Slide rack was placed in a water bath maintained at 95–98°C for 25 min and then was removed and allowed to cool at RT for 30 min. After cooling, slides were placed in 3% hydrogen peroxide (Fisher Scientific, Catalog No. H325-500) for 10 min. Immunostaining began with a blocking buffer (PBS with 5% normal horse serum and 3% Triton X-100) for 1 h at RT and was followed by overnight incubation at 37°C with rabbit anti-Myosin VIIa (Proteus Biosciences, Catalog No. 25-6,790) at 1:200 to identify hair cells. Secondary incubations consisted of two sequential 1 h incubations at 37°C with goat anti-rabbit IgG coupled to Alexa Flour dye (Thermo Fisher Scientific, Catalog No. A32732). As a counterstain, 4′,6-diamidino-2-phenylindole (DAPI, Thermo Fisher Scientific, Catalog No. D1306) was used. After staining, slides were immediately mounted. Images were captured using confocal microscopy (Zeiss LSM 880).

## Results and discussion

A temporal bone plug cutter was designed and manufactured through a collaboration with our institution’s machine shop. The design was inspired by the temporal bone plug cutter described by Nadol (1996). Three different iterations were made prior to the final design (Table 1). A technical drawing of the final design is provided in the Supplementary Figure S1. Important factors to consider include material type and cutting teeth design. Stainless steel was too malleable to withstand the force needed to penetrate through the skull base. Conversely, A2 tool steel was prone to brittle fractures without tempering. The final design utilized A2 tool steel with a Hardness Rockwell C (HRC) rating of 56/58 tempered at 600°F. Since an oscillating saw rotates bidirectionally, cutting teeth were designed without directionality. The initial version did not include an offset between teeth and so bone dust was not cleared effectively. Following iterations incorporated a 10° alternate offset which improved performance. The final design also added a small step-off from the cutting level to the main shaft to further aid in expulsion of bone dust. Shaft length was selected to ensure adequate penetration to reach inferior soft tissue attachments which tether the temporal bone. The base of the temporal bone plug cutter was designed to be compatible with the triangular shaft of the Stryker Autopsy Saw (Stryker Corp., Kalamazoo, Mich.). While this particular saw model has been discontinued, the base of the cutter can be easily modified to meet shaft dimensions of other commercially available oscillating autopsy saws.

**Table 1:** Temporal bone plug saw iterations.

Iteration	Material	Shaft length (mm)	Inner/Outer diameter (mm)	Cutting teeth design	Comments
1	Stainless steel	51	38/39	48 teeth, no offset	Inability to expel bone dust
2	Stainless steel	51	38/39	48 teeth, alternate 10° offset	Cutting teeth too malleable
3	A2 tool steel, HRC 56/58	51	38/40	45 teeth, alternate 10° offset	Steel not tempered leading to brittle fracture of the end of the shaft
4 (final design)	A2 tool steel, HRC 56/58, 600°F temper	51	38/40	45 teeth, alternate 10° offset, stepped-design	Design allows for cutting teeth to be at a step-off from main shaft allowing for improved flow of bone dust

HRC, Hardness Rockwell Scale C.

To date, we have collected more than 30 temporal bones using our custom-made temporal bone plug cutter. The average PMI was 52 h (range: 7–151). To assess decalcification times, eight fixed bones underwent a strict schedule of EDTA changes (twice weekly) and micro-CT imaging (weekly). Of these eight, four were routinely trimmed twice a month until the otic capsule was reached. Donor demographics, clinical histories, and PMIs of these eight bones along with their decalcification times are listed in Table 2. On average, it took the non-trimmed bone plugs 5.5 months (range: 5.1–5.8) to decalcify. The trimmed bones all decalcified faster, at 3.2 months. These bones required 5–6 trimming sessions to obtain a well-defined otic capsule. The remaining bones were either fixed and decalcified using the same techniques as above but with less stringent timing or were used as fresh specimens for device testing.

**Table 2:** Temporal bone plug donor demographics and medical history.

Donor	Age (years)	Sex	PMI (hours)	Hearing pathology	Cause of death	Trimmed	Left temporal bone decalcification time (months)	Right temporal bone decalcification time (months)
1	77	M	36	No known diagnoses	Acute hypoxemic respiratory failure	No	5.7	5.3
2	62	F	26	No known diagnoses	Acute hypoxemic respiratory failure	No	5.8	5.1
3	88	M	48	No known diagnoses	Large B-cell lymphoma	Yes	3.2	3.2
4	75	M	44	Left sided sudden sensorineural hearing loss	Acute on chronic heart failure following orthotopic heart transplant	Yes	3.2	3.2

PMI, post-mortem interval.

**Figure 5:**
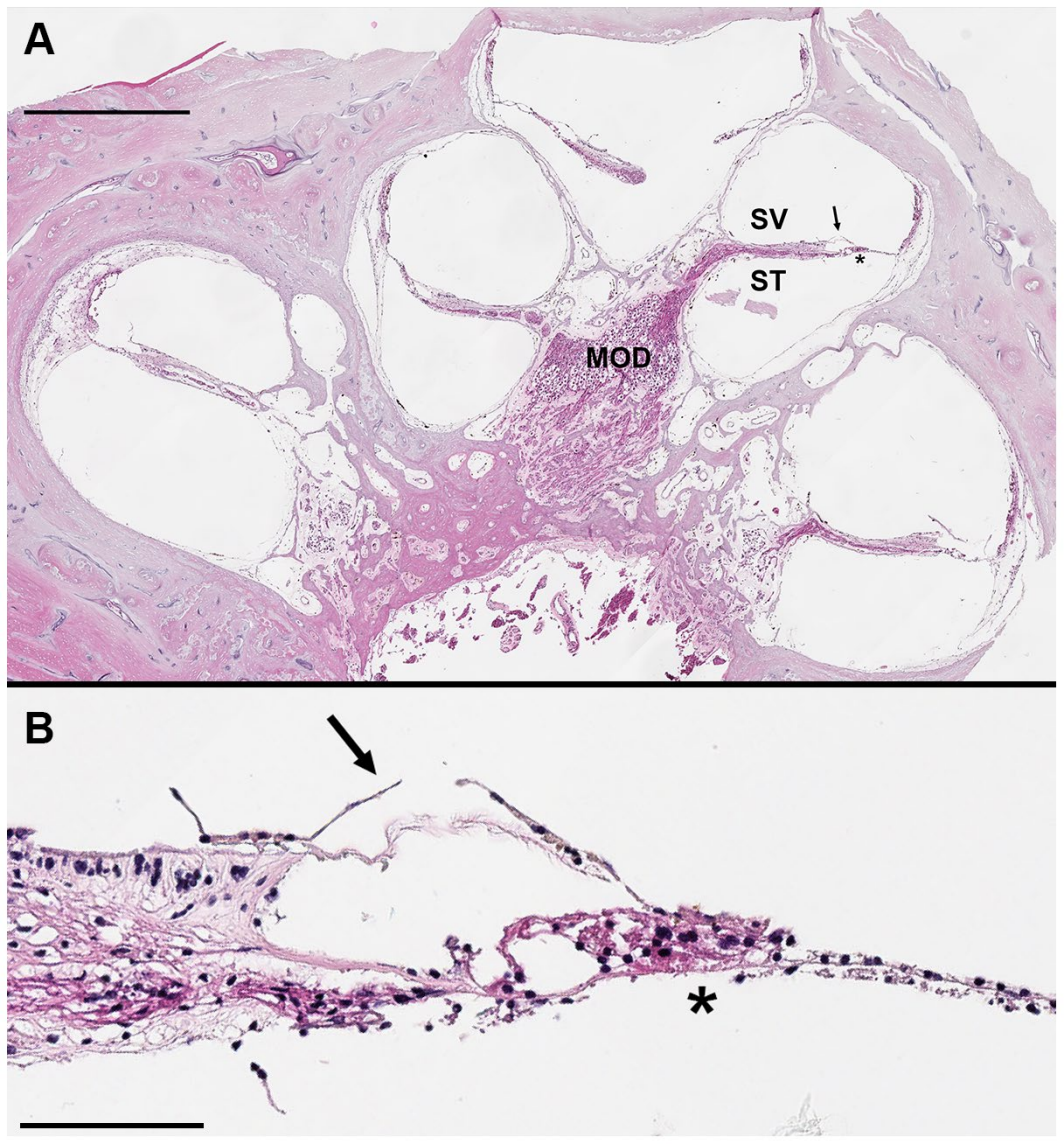
Hematoxylin and eosin stained mid-modiolar cochlear cross section. Donor bone was collected from a 75 years-old male at a 44 h post-mortem interval and was decalcified using ethylenediaminetetraacetic acid (EDTA), the microwave, and by trimming. The bone was embedded in paraffin and cut in 5 μm sections to achieve a mid-modiolar cross section. **(A)** Cochlear turns are seen spiraling around the modiolus (MOD) which contains spiral ganglion neuronal cell bodies and axons of the auditory nerve. Tectorial membrane (arrow) and basilar membrane (*) are clearly visualized, as are the scala vestibuli (SV) and scala tympani (ST). Scale bar = 1 mm. **(B)** Higher magnification image of organ of Corti. Hair cells and supporting cells present, but morphology limited likely due to long post-mortem interval. Tectorial membrane (arrow) and basilar membrane (*) visualized. Scale bar = 100 μm.

**Figure 6:**
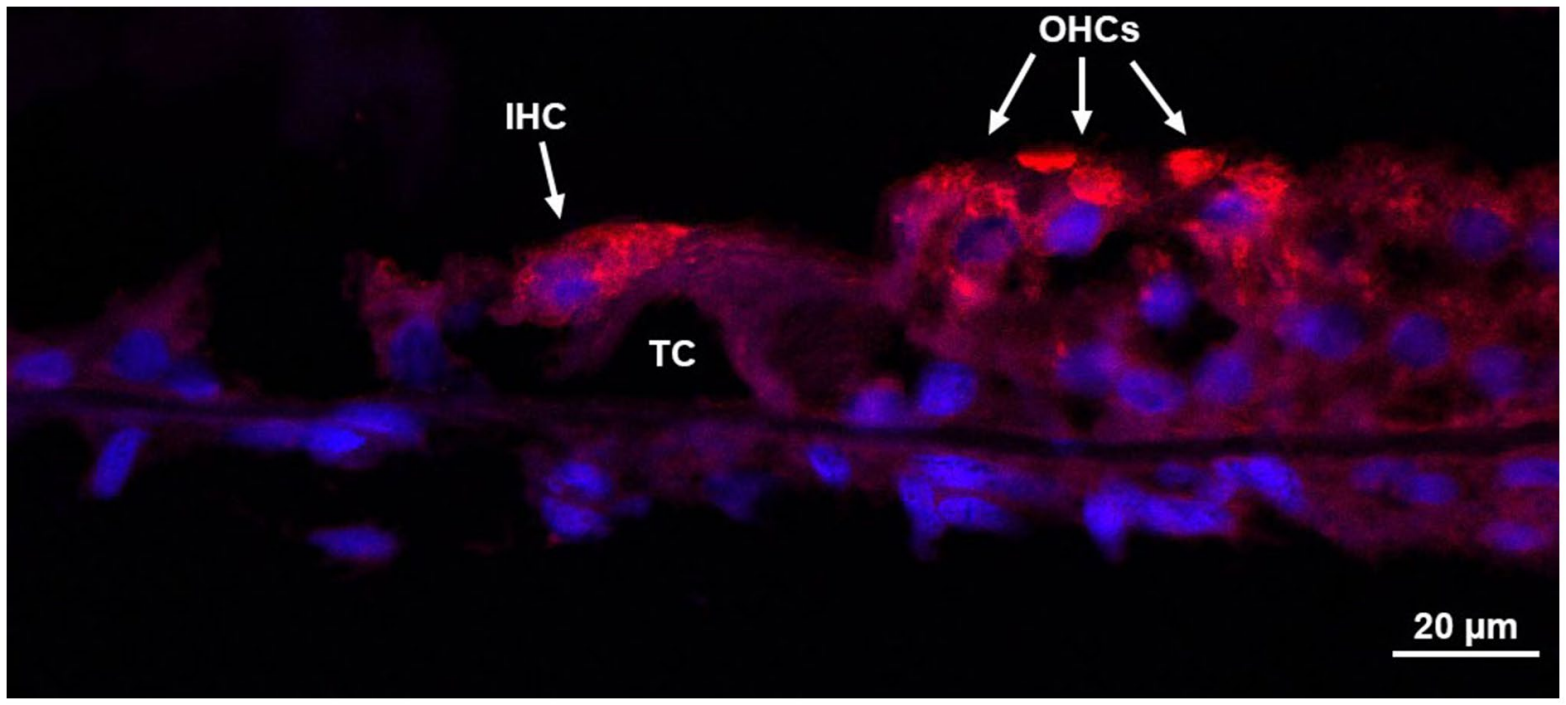
Immunohistochemistry of the organ of Corti. Donor bone was collected from an 87 years-old male at a 7 h post-mortem interval and was decalcified using ethylenediaminetetraacetic acid (EDTA), the microwave, and by trimming. The bone was embedded in paraffin and cut in 8 μm sections. Immunohistochemistry using anti-myosin VIIa antibodies of a mid-modiolar section revealed an organ of Corti with an intact inner hair cell, three outer hair cells, and the tunnel of Corti. IHC, inner hair cell; OHCs, outer hair cells; TC, tunnel of Corti.

H&E staining allowed for identification of mid-modiolar sections with intact organs of Corti so that adjacent sections could be used for immunostaining (Figure 5). H&E staining can locate anatomy of interest, including the SCs, utricle, saccule, osseous spiral lamina, basilar membrane, Reissner’s membrane, stria vascularis, spiral ligament, scala vestibuli, scala media, and scala tympani. However, not all fine morphology of structures was routinely preserved using this methodology in HTBs with PMIs greater than 24 h. Immunostaining of mid-modiolar sections revealed preservation of the basilar membrane, tunnel of Corti, cell nuclei, inner hair cells, and outer hair cells (Figure 6).

### Approach to decalcification

A major hindrance to efficient HTB research is the long decalcification time required prior to histopathological analysis. The standard EDTA decalcification approach used for morphological study can take up to 9 months (Lopez et al., 2016). Alternative decalcifying agents have been trialed, but they can lead to poor preservation of cytoarchitecture and immunogenicity (Ghosh et al., 2020). Microwave decalcification with EDTA has proven to accelerate decalcification time while maintaining structural morphology and tissue antigenicity (Keithley et al., 2000; Cunningham et al., 2001). Early attempts to incorporate the microwave were time consuming as it required regular calibration and identification of ‘cool spots’ to prevent overheating of the specimen (Keithley et al., 2000; Cunningham et al., 2001). The latest microwave models no longer require manual adjustments to prevent specimen overheating. Instead, a set-point temperature is maintained by a water cooling pump controlled by the microwave microprocessor. Since use is no longer restricted to a single spot in the microwave, we were able to decalcify as many as six samples in parallel, all maintained at 25°C. Our full-sized temporal bone plugs were decalcified in less than 6 months with minimal effort, a process that is approximately three months faster than standard EDTA decalcification.

Additional strategies can be employed to further accelerate decalcification. Frequent EDTA changes can ensure adequate reagent availability for calcium chelation but is more labor intensive; a higher EDTA concentration can overcome this limitation. Routine trimming of bone decreased decalcification time and required only a scalpel or rongeur. Immediate drilling to the level of the otic capsule followed by microwave EDTA decalcification may be the quickest method. Any combination of these approaches can be implemented as needed based on resource availability, personnel knowledge, and experimental constraints.

### Histopathologic analysis

The gold standard for evaluation of temporal bone pathology has long been histopathologic analysis with light microscopy. As availability of antibodies to inner ear antigens continues to rise, immunostaining has also been established as a reliable analytical approach. Different techniques for immunohistochemical analysis of HTBs have been described in depth and can vary based on selection of embedding media (Lopez et al., 2016).

Traditional HTB processing, performed primarily in specialized otopathology laboratories, utilizes celloidin embedding. Celloidin is known to have the best morphological preservation of all tested embedding media but was originally thought to suffer from poor immunogenicity (O’Malley et al., 2009a). As techniques for celloidin removal and antigen retrieval have improved, immunostaining has been successful even in archived celloidin embedded HTB sections (O’Malley et al., 2009a, b). However, celloidin embedding is performed over several months and requires complex technical knowledge and specialized equipment for proper sectioning.

An alternative embedding medium, and one that is commonly used in histological study of other organ systems, is paraffin. Paraffin embedding has been shown to preserve fine morphology of the inner ear in bones with short PMIs but is more prone to diminishing structural integrity compared to celloidin (Cunningham et al., 2001; O’Malley et al., 2009a; Takahashi et al., 2010). However, paraffin is an ideal medium for immunostaining, which is thought to be a result of the high processing temperatures improving antigen–antibody recognition (O’Malley et al., 2009a). From a resource perspective, paraffin embedding requires equipment and reagents found in most pathology departments. Moreover, the techniques associated with paraffin embedding are amenable to efficient learning even for those without prior experience. Of note, even the best processing strategies cannot overcome long PMIs with prolonged autolysis of tissue. Close collaboration with pathology colleagues is paramount to obtaining samples with short PMIs under rapid autopsy protocols (Bacon et al., 2020; Hooper, 2021).

### Downstream applications

Numerous advances in the clinical practice of otology can be attributed to the study of HTBs. Traditional histopathological analysis has been pivotal to the understanding of common otologic diseases (Hallpike and Cairns, 1938; Mudry, 2006) and has helped to refine surgical technique in procedures such as the placement of a cochlear implant (Richard et al., 2012; Nadol et al., 2014). As adoption of immunohistochemistry has increased, new insights into the mechanisms of hearing loss have also been uncovered. For instance, immunostaining for four different inner ear markers in parallel revealed that cochlear synaptopathy may be an important contributor to presbycusis (Viana et al., 2015). While these methods form the backbone of HTB research, alternative approaches have pushed the boundaries of what can be learned from each specimen. DNA retrieval from archived samples revealed the presence of varicella-zoster virus in the geniculate ganglion in patients with Ramsey-Hunt syndrome, confirming the etiology of the condition (Wackym, 1997). Additionally, applying Sanger sequencing to extracted DNA has helped correlate otopathological findings to a variant in the DFNA5 gene, a now known mutation which causes sensorineural hearing loss (Nadol et al., 2015). Using inductively coupled plasma mass spectrometry, it was demonstrated that the human cochlea can retain cisplatin, a known ototoxic chemotherapeutic agent, for years after treatment (Breglio et al., 2017). Coupling this approach with laser ablation revealed localized cisplatin accumulation in the stria vascularis, suggesting that the drug’s ototoxic effects are due to hyperaccumulation as opposed to hypersensitization (Breglio et al., 2017). Furosemide, another known ototoxic agent, was shown to cause edema and cystic changes in the stria vascularis, collapse of the Reissner’s and tectorial membranes, and diffuse loss of hair cells (Santos and Nadol, 2017). HTBs have also served as effective models for evaluating therapeutic drug delivery to the middle and inner ear (Early et al., 2021; Veit et al., 2022). Multifunctional nanoparticles have been shown to cross the round window membrane and penetrate into the sensory hair cells and nerve fibers of the cochlea (Roy et al., 2012). Additionally, bisphosphonate, a potential therapeutic agent in otosclerosis, was shown to reach the apical turn of the cochlea when delivered via the oval window (Kang et al., 2016). These studies highlight the utility of HTBs in guiding pharmacological development prior to clinical testing. Additional exciting research opportunities are opening up when procuring living human inner ear tissues from deceased organ donors immediately after collection of vital organs for transplantation (Vaisbuch et al., 2022).

**Figure 7:**
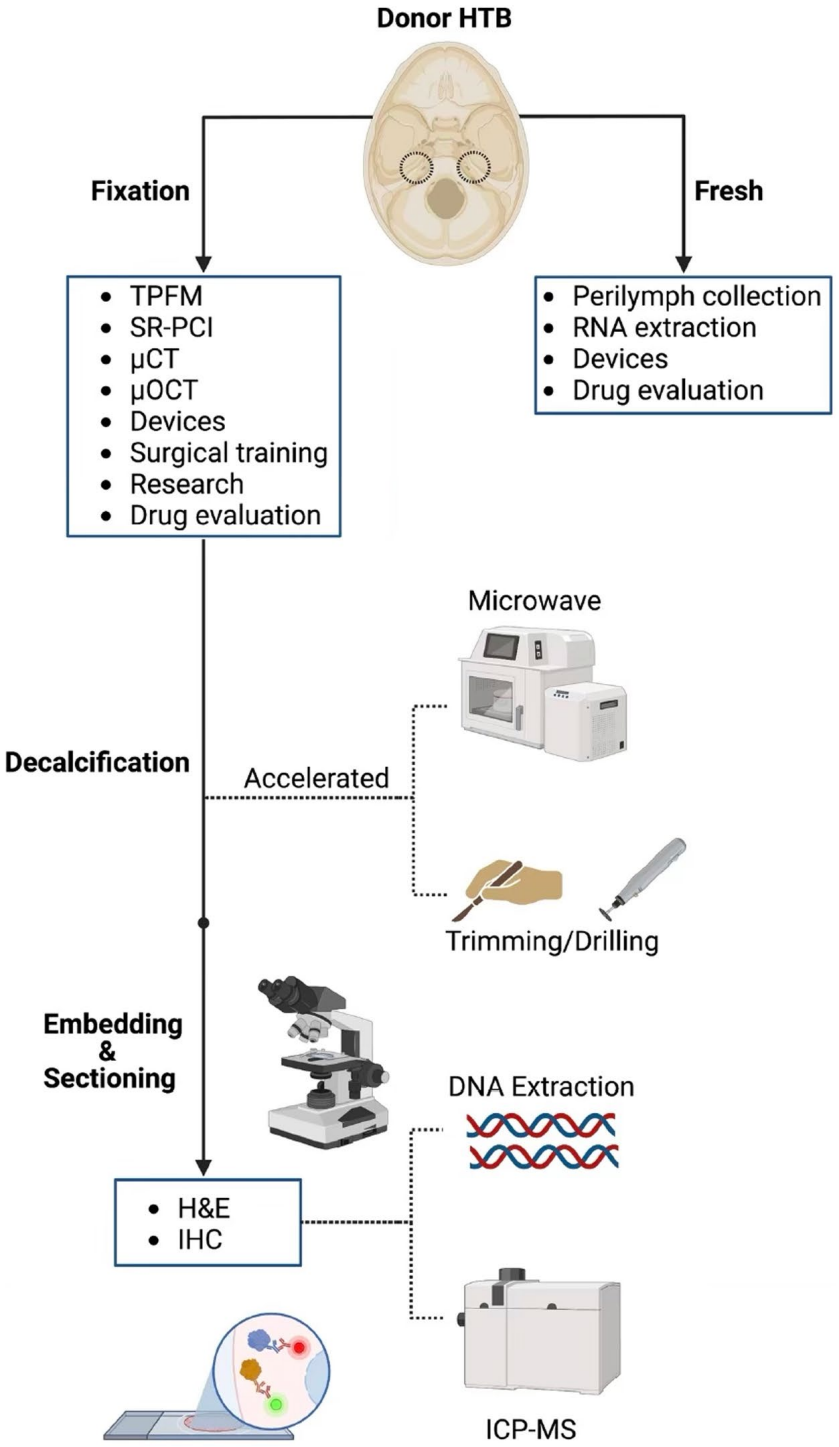
Processing pipeline and downstream applications for human temporal bones. The different processing steps for human temporal bone (HTB) analysis are displayed along with potential applications at each step of the pathway. TPFM, two-photon fluorescence microscopy; SR-PCI, synchrotron radiation phase contrast imaging; μCT, micro-computed tomography; μOCT, micro-optical coherence tomography; H&E, hematoxylin and eosin; IHC, immunohistochemistry; ICP-MS, induction coupled plasma mass spectrometry.

A drawback of cutting HTBs in sections is that the three-dimensional structure is not preserved. Novel imaging techniques have been investigated to overcome this limitation. Two-photon fluorescence microscopy (TPFM) was able to reveal cellular and subcellular structures in an unstained, non-sectioned, and calcified HTB when imaged through a cochleostomy (Iyer et al., 2021). Additionally, synchrotron-radiation phase-contrast (SR-PCI) imaging was able to reveal sensory cells and nerve fibers when used in a similar specimen (Iyer et al., 2018). Employing these techniques can be especially useful when validating new imaging devices. A recent example involves the efforts to incorporate micro-optical coherence tomography (μOCT) into a flexible endoscope format for inner ear imaging (Iyer et al., 2021). The device was capable of visualizing the three-dimensionally intact human cochlea at a resolution of up to 2 μm, allowing for clear distinction between damaged and intact organs of Corti (Iyer et al., 2021). Verifying accuracy of imaging with traditional histology can be time prohibitive, and therefore, TPFM and SR-PCI can serve as useful alternatives. Reversible iodine staining with micro-CT has also been shown to provide high-resolution intracochlear images and may be more accessible to researchers (Bommakanti et al., 2022). Beyond imaging applications, structurally intact fixed and fresh HTBs have been used in the development of a micro-needle device for controlled perilymph extraction (Early et al., 2019) as well as a fully implantable cochlear implant (Yip et al., 2015). While the applications of HTBs are numerous and will continue to grow as new technologies develop, it is imperative to consider a HTB’s PMI when choosing an application (Figure 7).

### Limitations

Our study has a few limitations. The creation of a temporal bone plug sawblade may require access to an institutional machine shop or a private vendor which can be time-consuming and costly. The outlined approach also requires access to an autopsy program, preferably one that is capable of conducting rapid autopsies if HTBs with short PMIs are required. Additionally, while all efforts were made to be cost-conscious in our approach to facilitating procurement and processing of HTBs, the study of these specimens inherently requires high direct and indirect costs that are best addressed with government and institutional funding.

## Conclusion

There is no shortage of downstream applications for which HTBs can be used in auditory research. Prior efforts have demonstrated their utility across the spectrum of diagnosis and treatment of otologic disease. Procurement and processing of HTBs can be technically challenging and time consuming but these challenges can be addressed using our detailed approaches. We have provided both methods and technical resources to facilitate the ongoing study of HTBs. Collection of all available specimens along with associated clinical information is recommended. A processing approach should be chosen based on resource availability and experimental requirements.

## Data availability statement

The raw data supporting the conclusions of this article will be made available by the authors, without undue reservation.

## Ethics statement

The studies involving humans were approved by Stanford University Institutional Review Board. The studies were conducted in accordance with the local legislation and institutional requirements. The human samples used in this study were acquired from a by-product of routine care or industry. Written informed consent for standard autopsy was obtained from the participants’ next of kin in accordance with the national legislation and institutional requirements.

## Author contributions

KS conceived the study and supervised all aspects of the work. VS and KS designed the study. VS, NK, LM, JM, XM, DR, and JH collected data. VS and MS designed the temporal bone plug sawblade. VS and NK wrote the manuscript. All authors contributed to the article and approved the submitted version.

## Supplementary material

The Supplementary material for this article can be found online at: https://www.frontiersin.org/articles/10.3389/fnins.2023.1242831/full#supplementary-material and https://figshare.com/s/a357a9a7f72a0eda95a1
