# Supplementary material for: Mortui vivos docent: a modern revival of temporal bone plug harvests
Source: Front Neurosci. 2023 Oct 11;17:1242831. doi: 10.3389/fnins.2023.1242831 (PMC10598599; doi:10.3389/fnins.2023.1242831)
Supplement: Supplementary file 1 [file Data_Sheet_1.DOCX]

Supplementary Material

**Supplementary Figures**

**Supplemental Figure 1. Technical drawing of temporal bone plug sawblade**

A technical drawing is provided as a guide to facilitate creation of a temporal bone plug sawblade either through an institutional machine shop or private vendor. The base of the sawblade can be easily modified to fit chosen autopsy saw.

**Supplementary Videos**

**Supplemental Video 1. Extraction of human temporal bone with bone plug saw**

This video depicts the process of extracting bilateral human temporal bone plugs from autopsy donors.

**Supplemental Video 2. Drilling of temporal bone to otic capsule**

This video illustrates the process of drilling a whole temporal bone resected en bloc down to the otic capsule. Depending on skill level, this process can take anywhere from 3-5 hours. The otic capsule can then be placed in ethylenediaminetetraacetic acid (EDTA) and in the microwave for final decalcification.

**Supplementary Materials and Methods**

**Techniques & Tips for Trimming of Temporal Bone to Otic Capsule**

1. The light of the microscope can dry out the bone. It is important to periodically dip the bone into ethylenediaminetetraacetic acid (EDTA) or saline while trimming to ensure hydration and prevent inadvertent removal of more bone than anticipated, especially when close to the otic capsule.
2. Use sharp, complete cuts made similarly to how wood is whittled to remove the bone and avoid pulling at or peeling off partially cut slices of decalcified bone to prevent excess removal or violation of important structures. Unlike in live tissue, decalcified tissue does not inherently respect planes of important structures.
3. When starting a trimming session, start with the fine work such as outlining semicircular canals (SCs) and the cochlea while the blade is still sharp and then move on to grosser work as the blade dulls. This forces one to define important structures first to avoid accidental injury. Alternatively, frequently switch out blades to maintain sharpness.
4. Decalcified bone tends to have a more white, translucent color while calcified bone has a more yellow hued tinge and feels slightly rougher. As one approaches the otic capsule and encounters calcified bone (yellow tinge), one should stop and return the bone to EDTA. During the next session, if more decalcified bone is visible, more can be trimmed.
5. One of the last remaining portions of calcified bone tends to a be a very large, triangular wedge of the mastoid. Decalcification time can be decreased by whittling down this area to a spike during every trimming session. Once at a calcified spike, use a series of increasingly fine rongeurs to cut it down into several pieces. Of note, observers may want to consider wearing eye protection as these bone fragments may fly. Even a few snips of the bone in this area can reduce decalcification time.
6. Another area of persistent calcified bone is around the SCs, specifically in the area beneath their arches. Decalcification can be expedited by creating connections between the bone beneath the arches of the canal loops or by removal of any decalcified portions of bone around that area to allow for greater EDTA penetration. To visualize the connection, one may need to remove bone dust using 0.5mm tweezers. For beginners, we recommend starting with the superior SC, as the arcuate artery runs through this location and creates a natural connection that can guide the carving beneath the canal of this arch. However, if studying SC pathology, it may be best to wait for decalcification of this area to occur via EDTA and the microwave to prevent accidental penetration into the SCs.
7. On a similar note, one may also want to consider leaving the decalcified bone in the area of the labyrinthine segment of the facial nerve between the cochlea and the SCs while much trimming remains to be done for the rest of the bone, as removing it can make the connection between the two unstable and at risk for fracture through the vestibule or separation. One can either wait until this area is decalcified via EDTA and the microwave or remove as one of the last steps.
