# Supplementary material for: Mortui vivos docent: a modern revival of temporal bone plug harvests
Source: Front Neurosci. 2023 Oct 11;17:1242831. doi: 10.3389/fnins.2023.1242831 (PMC10598599; doi:10.3389/fnins.2023.1242831)
Supplement: Supplementary file 2 [file Image_1.PDF]

Profile

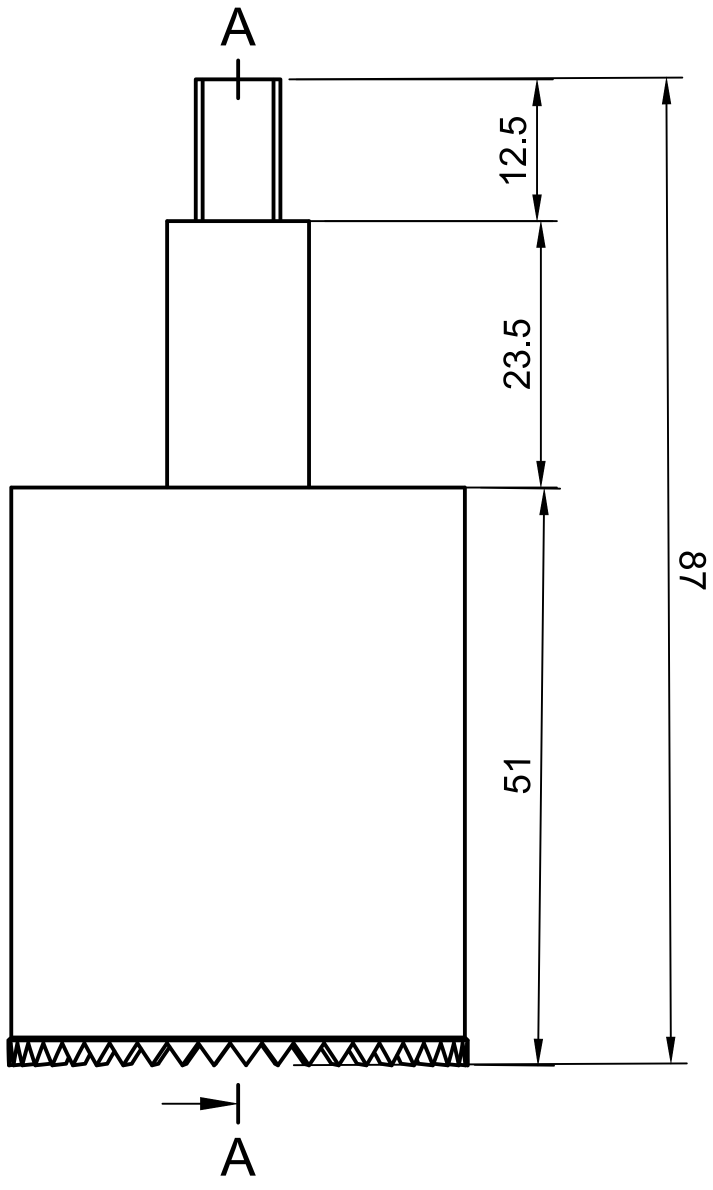

A: Slice Image

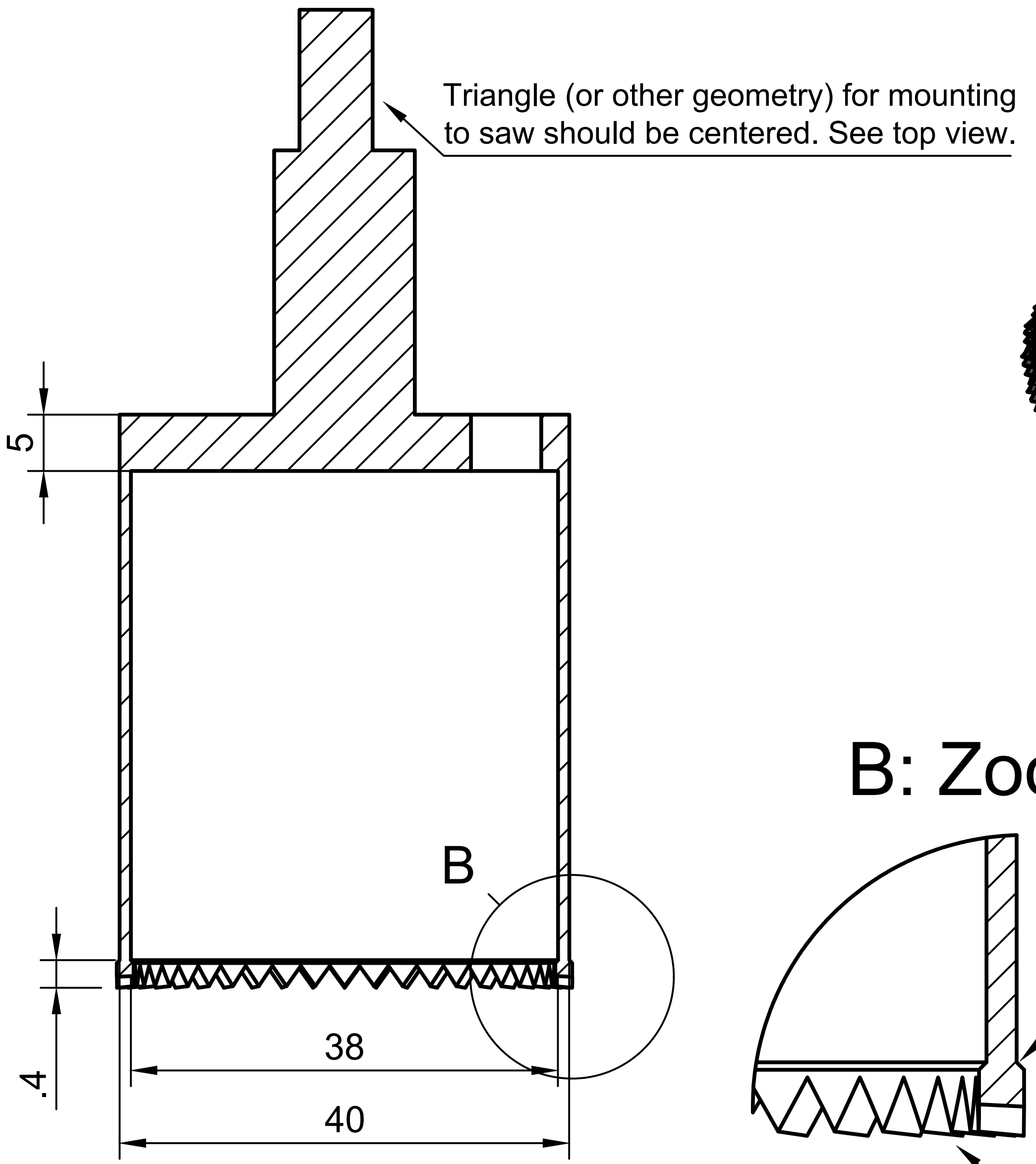

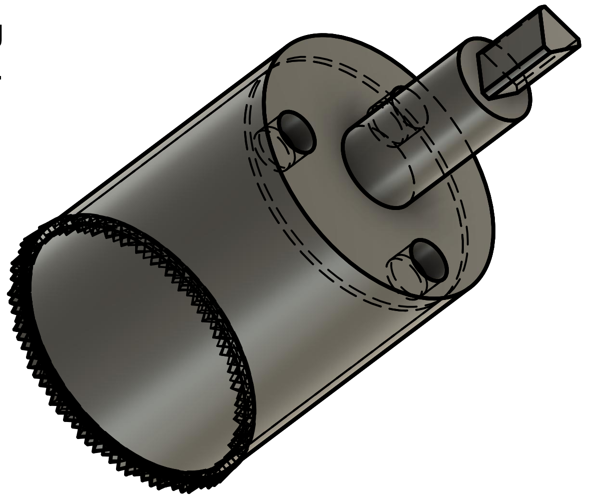

B: Zoom

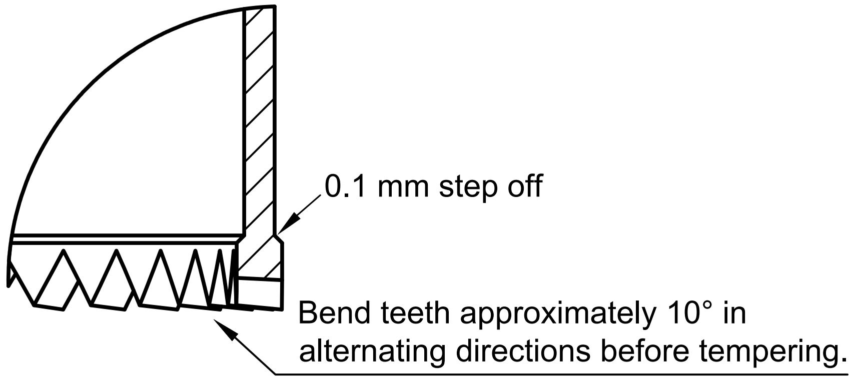

Top View

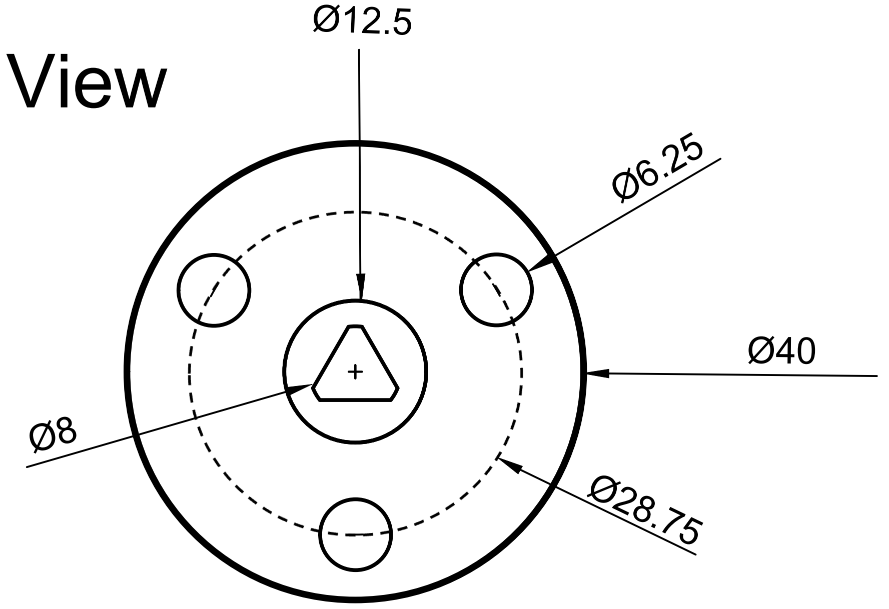

All units in mm

Dept.	Technical reference		Approved by		
		Document type	Document status		
		Title<br>Bone Plug Saw Blade	DWG No.		
			Rev.	Date of issue	Sheet<br>1/1
